# Reporting community involvement in autism research: Findings from the journal Autism

**DOI:** 10.1177/13623613241275263

**Published:** 2024-09-06

**Authors:** Diana Weiting Tan, Laura Crane, Tori Haar, Melanie Heyworth, Rebecca Poulsen, Elizabeth Pellicano

**Affiliations:** 1 Macquarie School of Education, Macquarie University, Australia; 2 School of Psychological Science, The University of Western Australia, Australia; 3 Autism Centre for Education and Research (ACER), Department of Disability, Inclusion and Special Needs, School of Education, University of Birmingham, UK; 4 Centre for Research in Autism and Education, IOE, UCL’s Faculty of Education and Society, University College London, UK; 5 Reframing Autism, Australia; 6 Department of Linguistics, Faculty of Medicine, Health and Human Sciences, Macquarie University, Australia; 7 Department of Clinical, Education and Health Psychology, University College London, UK

**Keywords:** autism, community involvement, participatory research, patient and public involvement, stakeholders

## Abstract

Participatory methods are having sweeping effects on research across the globe. To facilitate transparency around these methods, *Autism* introduced mandatory reporting of community involvement in January 2021. Here, we sought to determine the impact of this policy. To do so, we – a team of Autistic and non-autistic researchers – searched for articles published in *Autism* in 2019, 2 years before policy implementation, and in 2022, 1 year after. We read 283 eligible articles and double coded each article for general information and details of community involvement. We found a fivefold increase (10.3% pre-implementation vs 54.5% post-implementation) in the reporting of community involvement. Most articles reported involving community members distally, in consulting roles, with researchers retaining full decision-making power. Furthermore, most community involvement was reported in more applied than basic research. A significant minority of papers (17%), however, could not be coded, owing to insufficient or vague information. While this increase in the reporting of community involvement is encouraging, more efforts need to be directed towards (1) improving the transparency in the reporting of community involvement and (2) increasing the frequency with which community members, especially Autistic people, share decision-making power equally with researchers – or even assume control of the research process.

**Lay abstract:**

There has been a growing push for the Autistic and autism communities to be more actively involved in autism research. From January 2021, the journal *Autism* made it a rule for authors to report whether they involved community members in their work; and if they did, how they had done so. In this study, we wanted to see how this new rule has changed things. Our team of Autistic and non-autistic researchers read all 283 articles published in *Autism* in 2019, about 2 years before the rule was in place, and in 2022, about 1 year after. We recorded what each article was about and how the community was involved. We found there was an increase in how often articles talked about community involvement – from about 10% before the rule to over 50% after. Most of these studies, however, only involved community members giving advice, with the researchers making most decisions about the research. This was especially true for applied research (like wellbeing) rather than basic science (like causes of autism). Also, some of these articles were unclear or did not give enough information for us to understand how the community was involved. This tells us that while it is promising that more community involvement is reported, researchers need to describe this involvement more clearly. It is also important for community members to have a bigger say in research by sharing power with the researchers or even leading the research themselves.

Participatory approaches are having major effects on research across the globe, in disciplines ranging from agriculture and environmental science to health-related research and research with minority communities (Macaulay, 2017). Such approaches challenge conventional research methods, in which research is typically done *to*, *on* or *for* individuals, and are part of a wider movement to democratise research (Lloyd & White, 2011). Researchers adopting participatory approaches aim to work together *with* individuals outside academia, drawing on the ‘practical wisdom’ of community members to shape the research process. Doing so can have a dramatic impact on the research agenda and on the research itself, strengthening ‘the rigour, relevance and reach of science’ (Balazs & Morello-Frosch, 2013, p. 10) and ensuring more ‘socially robust’ research (Turnhout et al., 2020, p. 16). The eventual concrete benefits of participatory approaches, especially those that attempt to form equitable partnerships between researchers and non-researchers, also go beyond epistemic ones. Such research can be part of endeavours to redress long-standing power imbalances in the production of knowledge (Beresford, 2003; Rose, 2013) by involving those who have the greatest stake but who have previously been excluded.

The increasing tendency to provide opportunities for non-researchers to participate in the design and delivery of research – which some have called a *participatory zeitgeist* (Palmer et al., 2019) – is also evident within autism research. A decade ago, Autistic people were rarely involved in the decision-making processes that shape research or its application (Jivraj et al., 2014; Pellicano & Stears, 2011) – and the vast majority of researchers gave no impression of being particularly enthusiastic about them being co-producers of research (Pellicano et al., 2014a). Yet, following concerted calls from Autistic scholars, neurodiversity activists, public policy stakeholders and others (e.g. den Houting, 2019; Milton, 2014; Robertson, 2010), there is now a growing movement towards working with Autistic people, family members and their supporters, where community members are actively involved in what kind of research is done, how it is done, how research results are interpreted and how the findings are used (Fletcher-Watson et al., 2019; Nicolaidis et al., 2019; see Tan et al., 2024 for review). Autistic academics are, of course, increasingly leading their own research, too (e.g. Bertilsdotter Rosqvist et al., 2023; Botha, 2021; Poulsen et al., 2022). Furthermore, the commitment to participatory approaches from funding bodies has already generated a shift in the funding landscape, at least in some jurisdictions, bringing research funding more in line with community priorities (den Houting & Pellicano, 2019).

We believe this momentum for participatory autism research is encouraging. Yet, early analysis suggests that autism research that involves community partners in some or all aspects of the research does not always live up to its stated objectives. For example, one study that elicited non-autistic academics and community partners’ experiences of involvement in participatory autism research revealed less than encouraging results (den Houting et al., 2021). Although respondents were enthusiastic about the potential of participatory autism research, only 1 participant (a community partner) out of 79 explicitly commented on the importance of addressing power differentials between non-autistic researchers and community members. Yet such power dynamics remained entrenched in many of den Houting et al.’s (2021) respondents’ experiences of research, with community partners’ involvement often confined to consulting (Figure 1 and Table 1) rather than collaborating on research projects. As one participant explained, ‘we have been asked by university personnel to *co-lead* projects in order to comply with funding guidelines, but then we have not always been invited to meetings to plan the research’ (p. 157, emphasis added).

**Figure 1.**
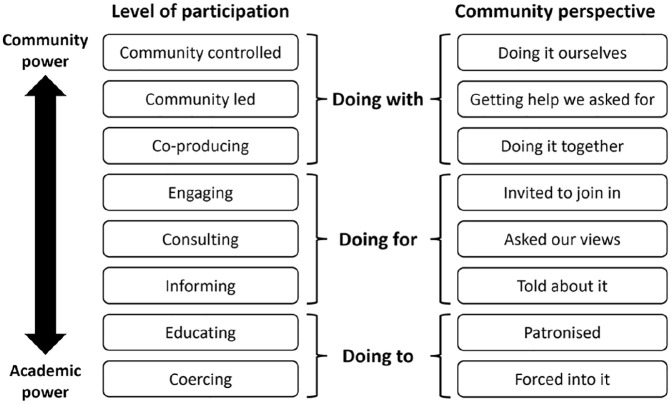
Adaptation of Arnstein’s (1969) ladder of participation reproduced with permission from den Houting et al. (2021).

**Table 1.** Characteristics of each of the eight rungs of the participatory ladder based on Arnstein (1969).

Level of participation	Characteristics
Community controlled	Community members have full decision-making and negotiation power over all aspects of the research. Participation at this level signifies the highest level of power redistribution, with community members holding all the decision-making power.
Community led	Community members have dominant decision-making authority over all aspects of the research. Participation at this level signifies a high level of power redistribution, with community members holding more decision-making power than researchers.
Co-producing	Community members and researchers share planning and decision-making responsibilities across all aspects of the research. Participation at this level signifies some level of power redistribution, with community members holding equal decision-making power as researchers.
Engaging	Community members are invited to advise on research, but researchers retain the right to determine the legitimacy or feasibility of the advice. The breadth of participation is also limited to only a few aspects of the research (e.g. reviewing procedure and participant-facing materials). Participation at this level is tokenistic with researchers retaining more decision-making power than community members.
Consulting	Community members are invited to voice their opinions, but it is not clear whether researchers have taken them into account. The breadth of participation is also limited (e.g. only reviewing participant-facing materials). Participation at this level is tokenistic with researchers retaining most decision-making power.
Informing	Community members are informed about decisions made in research, but there is no channel for feedback and no power for negotiation. Participation at this level is tokenistic with researchers retaining full decision-making power.
Educating	Community members are educated about research to ‘correct’ their ‘misconceptions’ about research, rather than being genuinely involved. There is no participation at this level.
Coercing	Community members are misled and manipulated into being involved in research for the purpose of optics. There is no participation at this level.

In this study, we have defined community members as individuals from the Autistic community (lay Autistic people and Autistic researchers) or the broader autism community (family members of Autistic people and professionals). This approach reflects current understanding in the field and acknowledges the non-academic and lived experiences that community members bring. However, there is a need to shift the power in shaping research processes to Autistic community members, particularly lay Autistic people outside formal academic institutions (see section ‘Discussion’).

The lack of effective and equitable partnership between non-autistic academics and community partners may be a result of often vague, confused and unambitious understandings of participatory research. Evidence for this possibility comes from den Houting et al.’s (2021) academics’ definitions of participatory research, which ranged from ‘any participation, directly or indirectly, relating to the autism research’, to processes of consultation (‘being given ample opportunity to feed back to the researchers’; ‘being involved in an advisory capacity’) and, far less commonly, shared power in research decision-making. Similarly, in Pickard et al.’s (2022) study with early-career and established autism researchers, many interviewees expressed concerns about participatory research being a ‘very broad concept’ and were confused about what does or does not ‘count’ as participatory research (Hollin & Pearce, 2019).

The variability in researchers’ understanding of participatory research is perhaps unsurprising. Participatory research is an umbrella term that refers to a range of research methods that employ inclusive and community-engaged practices (Cargo & Mercer, 2008). Participation itself can take many forms, ranging from consultation through to collaboration (or ‘co-production’) and community-controlled research (Arnstein, 1969). This flexibility is important because the extent and nature of community involvement may depend on the topic, the research question, the methods and the resources available. Nevertheless, autism researchers’ conflicting understanding of what participatory research is, and their lack of knowledge around the role of power in shaping its processes and outcomes, is concerning. Inaccurately labelling projects as ‘participatory’ can have detrimental effects on community’s trust in autism research (Michael, 2021; Nicolaidis et al., 2019). More worryingly, doing participatory research in a superficial or tokenistic way can silence community members, by failing to engage fully with their experiential expertise and its role in knowledge production (so-called ‘participatory injustice’; Fricker, 2007; Patin et al., 2021). Such issues are not unique to autism research (Turnhout et al., 2020; Williams et al., 2020).

One way to enhance participatory approaches is to encourage researchers to be transparent about reporting the extent and nature of community involvement in their research. In 2015, *The British Medical Journal (BMJ)*, in consultation with community members, launched its patient partnership strategy, which required authors to include a Patient and Public Involvement (PPI) statement in their methods section in all research articles. It was hoped that such mandatory reporting ‘could have a myriad of extremely positive benefits’, including encouraging researchers to adopt participatory methods in their work, to reflect upon patients’ involvement in, and impact on, the research, and to reduce research waste (BMJ, 2018). The highly-regarded mental health journal, *Lancet Psychiatry*, has since followed suit (Davis et al., 2024).

Subsequent evaluation of this radical approach revealed that this hope was at least partly borne out (Price et al., 2018). One year before implementation of mandatory reporting of PPI, in 2014, only 1 of 189 articles (0.1%) published in *The BMJ* reported PPI activity. One year after its implementation, there was a 10-fold increase, with 16 of 152 published articles (11%) reporting PPI. That said, the frequency of PPI was low both before and after implementation, suggesting either a lack of uptake of participatory approaches more generally or limited willingness to describe unsuccessful attempts. Furthermore, while some authors described innovative approaches to PPI, others demonstrated a lack of awareness of what PPI means (Price et al., 2018; Weschke et al., 2023).

Clear, transparent and accurate reporting standards have also been suggested for participatory *autism* research (Hobson et al., 2023; Jivraj et al., 2014; Nicolaidis et al., 2019). To this end, and following *The BMJ*, one of the leading journals in autism research, *Autism*, introduced mandatory reporting of community involvement in January 2021 (see Supplementary Material for journal policy). Here, we sought to understand the impact of this approach. Specifically, we examined the nature and extent of community involvement in all articles published in *Autism* before (in 2019) and after (in 2022) the implementation of mandatory community involvement statements. Following Price et al. (2018), we asked the following research questions:

1. *Research Question 1 (RQ1).* Was there an increase in the reporting of community involvement following the journal’s implementation of the mandatory reporting requirement?
2. *Research Question 2 (RQ2).* Which community members were involved, and what was the degree of their involvement? Did this change over time?
3. *Research Question 3 (RQ3).* Which factors were associated with greater community involvement (e.g. country, research area, type of study, funding source)?

## Method

A protocol detailing the method of this study, including coding schemes, was pre-registered on the Open Science Framework (23 October 2023): https://osf.io/h5g4c/.

### Samples

We collected two samples of articles published in *Autism*. The first comprised articles published online between 1 January 2019 and 31 December 2019, approximately 2 years before the implementation of the journal’s reporting requirement in January 2021. The second sample consisted of articles published online between 1 January 2022 and 31 December 2022, approximately 1 year after policy implementation, to account for publication lag. Using PubMed, we identified 299 articles published during the stipulated time periods (2019: *n* = 123; 2022: *n* = 176).

#### Inclusion criteria

According to journal guidelines, community involvement statements are mandatory for research articles but optional for review articles. However, given that community members can be involved in both types of research, we included both research (*n* = 247; 87%) and review (*n* = 36; 13%) articles in our analysis. We excluded all other article types, including editorials and commentaries (*n* = 16; 5%).^1^

### Procedure

#### Coding

All eligible articles were coded using two coding schemes (see Tables S1 and S2) using a customised Google sheet integrated with Google forms. Using the first coding scheme, we extracted general information on each article, covering six categories: (1) the type of article (e.g. research article, review, etc.); (2) the type of research method (e.g. qualitative, quantitative, etc.); (3) the type of review method (e.g. narrative, systematic, etc.); (4) the country where the study was conducted, based on the corresponding author’s location;^2^ (5) research area (e.g. biology, lifespan, etc.) following the descriptions outlined in Interagency Autism Coordinating Committee’s 2016–2017 Strategic Plan (Office of Autism Research Coordination, 2019); and (6) the type of funder (e.g. governmental, industry, etc.).

The second coding scheme captured details about reported community involvement, including (1) the presence of a ‘Community Involvement Statement’; (2) the presence of community involvement in the research; (3) the types of community members involved (e.g. Autistic adults, parents of Autistic children, etc.); (4) the breadth of community involvement (e.g. choosing research methods, recruiting participants, etc., following den Houting et al., 2021); and (5) the level of participation, as defined by Arnstein (1969; for example, consulting, co-producing, etc.). For community involvement statements that were considered vague and unclear, these were classified as ‘insufficient information’ for categories 3–5. Any information that did not fit our pre-specified codes for categories 3–5 was entered verbatim. At the end of the coding process, the text was reviewed and, where possible, coded using the pre-specified scheme; when this was not possible, new categories were created (Tables S3 and S4). To facilitate data analysis and interpretation, we transformed our initial detailed coding scheme to a broad coding scheme for categories 3–5 (Tables S5 and S6).

All members of the research team, who themselves came from diverse backgrounds (Table S7), were involved in data extraction and coding. Every article was read in its entirety (including acknowledgements and contribution statements) and coded independently by two team members. None of the members coded articles on which they were an author. Discrepant codes were discussed and resolved by all members at monthly meetings. The majority of coding discrepancies occurred for five variables: research area (Cohen’s κ = 0.63, indicating substantial agreement according to Landis and Koch (1977)), presence of community involvement (Cohen’s κ = 0.79; substantial agreement), community members involved (Cohen’s κ = 0.87; near-perfect agreement), nature of the research activities (Cohen’s κ = 0.79; substantial agreement), and level of participation (Cohen’s κ = 0.83; near-perfect agreement).

#### Data analysis

We used R (R Core Team, 2017) and RStudio (RStudio Team, 2015) to compute descriptive statistics and visualise the data (see https://github.com/dianawtan/comm-inv-stmt for analysis codes). Inferential statistics (e.g. Chi-square tests) were not reported due to limited sample sizes, with >20% of most variables containing expected frequencies <5 (Howell, 2010).

### Community involvement statement

This study involved Autistic (T.H., M.H. and R.P.) and non-autistic (D.T., L.C. and E.P.) researchers working collaboratively. The study was initially conceptualised by non-autistic researchers (D.T., L.C. and E.P.). TH was then invited to the team and was involved in the development and testing of the coding schemes, followed by M.H. and R.P. All members of the team were involved in coding the articles, establishing consensus on the final codes, interpreting the data, and co-authoring this article (D.T. and E.P. wrote the initial draft which was reviewed by other authors). The research team met up online monthly to resolve discrepant codes and discuss study findings, their implications, and our discussion points. All members of the team – especially our Autistic researchers – perceive this effort to be an authentic research co-production. See Table S7 for an in-depth reflection on our participatory approach.

## Results

### Frequency of reporting

There was a fivefold increase in the proportion of studies reporting the presence of community involvement post-implementation of mandatory statements. Of the 116 eligible articles published in *Autism* prior to implementation of the mandatory community involvement statement, in 2019, only 12 (10%) described the presence of community involvement (11 research articles, 10%; 1 review, 1%). Of the 167 eligible articles published post-implementation, in 2022, 143 (86%) included community involvement statements – and just over half of these articles (*n* = 91, 54%) reported the presence of community involvement (one short report, 0.6%; 83 research articles, 50%; seven reviews, 4%; see Figure S1). The remaining (*n* = 76, 46%) stated that there was no community involvement in the research.

### Community members involved

Prior to policy implementation, most studies that reported community involvement included Autistic adults (*n* = 6; 50%) and parents of Autistic young people (*n* = 4; 33%). After implementation, while Autistic adults are still involved in a large proportion of these studies (*n* = 36; 40%), there was an increase in the representation of other groups, including Autistic researchers (*n* = 26; 29%) and clinicians/healthcare providers (*n* = 23; 25%). Involving Autistic young people, however, remained rare, with only one study in 2019 (8%) and two in 2022 (2%) reporting such involvement (see Table 2).

**Table 2.** Community members involved in articles that reported community involvement activities published before (2019) and after (2022) policy implementation.

Type of community members	*N (%)*	
	Before (*n* = 12)	After (*n* = 91)
***Autistic People***	***7 (58%)***	***51 (56%)***
Autistic adults	6 (50 ***%***)	36 (40 ***%***)
Autistic young people	1 (8 ***%***)	2 (2 ***%***)
Autistic researchers	2 (17 ***%***)	26 (29 ***%***)
***Families of Autistic People***	***5 (42%)***	***36 (40%)***
Parents of autistic adults	0	6 (7 ***%***)
Parents of autistic young people	4 (33 ***%***)	17 (19 ***%***)
Parents of autistic people (unspecified age)	0	3 (3 ***%***)
Family members (unspecified connection)	1 (8 ***%***)	4 (4 ***%***)
Researchers who are also parents of autistic people	0	7 (8 ***%***)
***Professionals***	***2 (17%)***	***32 (35%)***
Clinicians/Healthcare providers	1 (8 ***%***)	23 (25 ***%***)
Educators	1 (8 ***%***)	7 (8 ***%***)
Professionals (unspecified speciality)	0	5 (6 ***%***)
Researchers who are also professionals	0	2 (2 ***%***)
***Administrators***	***1 (8%)***	***6 (7%)***
Policymakers	1 (8 ***%***)	3 (3 ***%***)
Funders	0	1 (1 ***%***)
Patient/Advocacy organisations	0	2 (2 ***%***)
***Others***	***0***	***5 (6%)***
Community members (unspecified connection)	0	3 (3 ***%***)
Cannot be determined	0	2 (2 ***%***)

Categories and their associated values in bold and italics are based on the broad coding scheme (see Table S6). The remaining sub-categories and their associated values (neither in bold nor italics) are based on the detailed coding scheme (see Tables S2 and S6). As each study could involve more than one type of community member, percentages therefore do not add up to 100%.

### Level of participation

**Figure 2.**
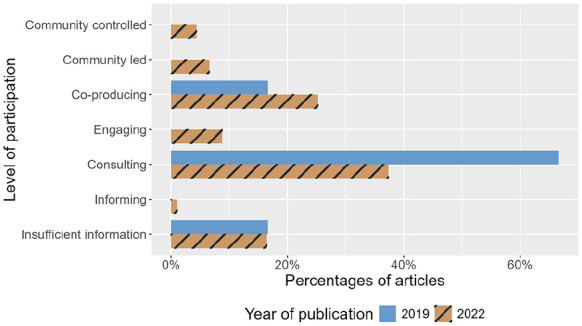
This bar chart shows the percentages of articles reporting community involvement across different levels of participation pre- and post-implementation of the mandatory reporting requirement. Bar colours and patterns indicate year of publication.

Before policy implementation, most research reported involving the community in consulting roles (*n* = 8; 67%). After implementation, there was an increase in community involvement extending beyond the consultative level (*n* = 34; 37%) to include co-produced (*n* = 23; 25%), community-led (*n* = 6; 7%) and community-controlled (*n* = 4; 4%) research. At both time-points, a significant proportion of articles did not provide sufficient information to allow for classification (2019: *n* = 2, 17%; 2022: *n* = 15, 17%; see Figure 2).

**Figure 3.**
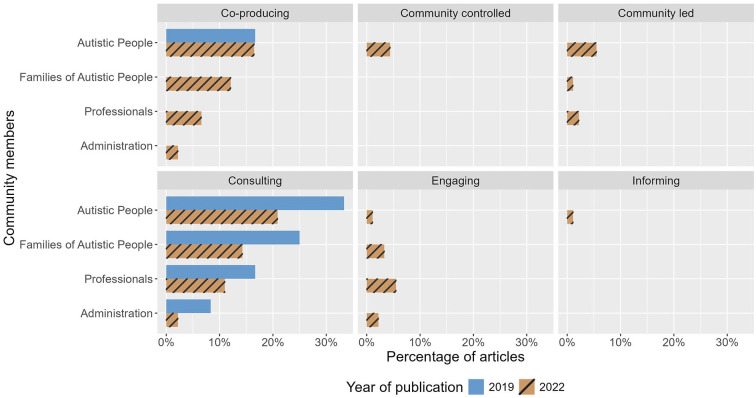
A panel of six bar charts showing the percentage of articles involving different groups of community members. Each chart represents each level of participation. Bar colours and patterns indicate year of publication.

We further examined the types of community members involved across different levels of participation (see Figure 3). At both time-points, Autistic people were most often consulted on studies that reportedly involved the community (2019: *n* = 4, 33%; 2022: *n* = 19, 21%), followed by co-producing studies (2019: *n* = 2, 17%; 2022: *n* = 15, 17%). Autistic people were also involved in community-controlled (*n* = 4, 4%) and community-led (*n* = 5, 5%) studies in 2022; both of which were absent from studies published in 2019. See Table S8 for a detailed breakdown of frequencies and percentages across different groups of community members and level of participation.

### Breadth of community involvement

Based on the broad coding scheme, before policy implementation, community members mostly contributed to study design (*n* = 8; 67%) and analysis (*n* = 5; 42%). After implementation, this trend persisted with a large proportion of studies involving community members in the design (*n* = 61; 67%) and analysis (*n* = 35, 38%) phases of research. Furthermore, the proportion of studies reporting community involvement across other aspects of research almost doubled over time, including conceptualisation (2019: *n* = 2, 17%; 2022: *n* = 27; 30%) and implementation (2019: *n* = 2, 17%; 2022: *n* = 29, 32%) – as well as those providing insufficient information (2019: *n* = 1, 8%; 2022: *n* = 17, 19%).

Both before and after policy implementation, more than two-thirds of studies involved the community in study design (Table 3). Closer examination of the breadth of involvement revealed, however, that community members were mostly involved in designing/modifying interview schedules and/or survey questions (2019: *n* = 4, 33%; 2022: *n* = 41, 45%) and less often in developing sampling procedures (2019: *n* = 1, 8%; 2022: *n* = 7, 8%). Nevertheless, post-implementation, there was a greater variety of activities in which community members were involved, with the most significant increases observed at the initial stages of the research process, including in background research (2019: *n* = 1, 8%; 2022: *n* = 22, 24%) and choosing research methods (2019: *n* = 1, 8%; 2022: *n* = 20, 22%).

**Table 3.** Breadth of community involvement based on the type of research activity involving community members before (2019) and after (2022) policy implementation.

Type of research activity	*N (%)*	
	Before (*n* = 12)	After (*n* = 91)
***Conceptualisation***	***2 (17%)***	***27 (30%)***
Developing community-based theories	2 (17 ***%***)	11 (12 ***%***)
Grant proposal writing	0	3 (3 ***%***)
Background research	1 (8 ***%***)	22 (24 ***%***)
Providing endorsement on project	0	1 (1 ***%***)
***Design***	***8 (67%)***	***61 (67%)***
Designing study (approach unspecified)	2 (17 ***%***)	28 (31 ***%***)
Choosing research method	1 (8 ***%***)	20 (22 ***%***)
Developing sampling procedures	1 (8 ***%***)	7 (8 ***%***)
Designing/Modifying interview schedules and/or survey questions	4 (33 ***%***)	41 (45 ***%***)
Providing inputs on public-facing materials	1 (8 ***%***)	2 (2 ***%***)
Developing/Adapting intervention	2 (17 ***%***)	6 (7 ***%***)
***Implementation***	***2 (17%)***	***29 (32%)***
Recruiting study participants	1 (8 ***%***)	18 (20 ***%***)
Implementing intervention	1 (8 ***%***)	8 (9 ***%***)
Collecting primary data	1 (8 ***%***)	17 (19 ***%***)
***Analysis***	***5 (42%)***	***35 (38%)***
Analysing data	2 (17 ***%***)	21 (23 ***%***)
Interpreting study findings	5 (42 ***%***)	30 (33 ***%***)
***Dissemination***	***3 (25%)***	***30 (33%)***
Writing reports/journal articles	2 (17 ***%***)	25 (28 ***%***)
Giving presentations at meetings/conferences	1 (8 ***%***)	3 (3 ***%***)
Disseminating findings (approach unspecified)	0	4 (4 ***%***)
***Insufficient information to determine***	***1 (8%)***	***17 (19%)***

Categories and their associated values in bold and italics are based on the broad coding scheme (see Table S5). The remaining sub-categories and their associated values (neither in bold nor italics) are based on the detailed coding scheme (see Tables S2 and S5). As each study could involve community members in more than one type of activity, percentages therefore might add up to more than 100%.

### Factors associated with community involvement

We examined four factors that might be associated with community involvement, including (1) country, (2) research areas, (3) research methods and (4) funders.

#### Country

**Figure 4.**
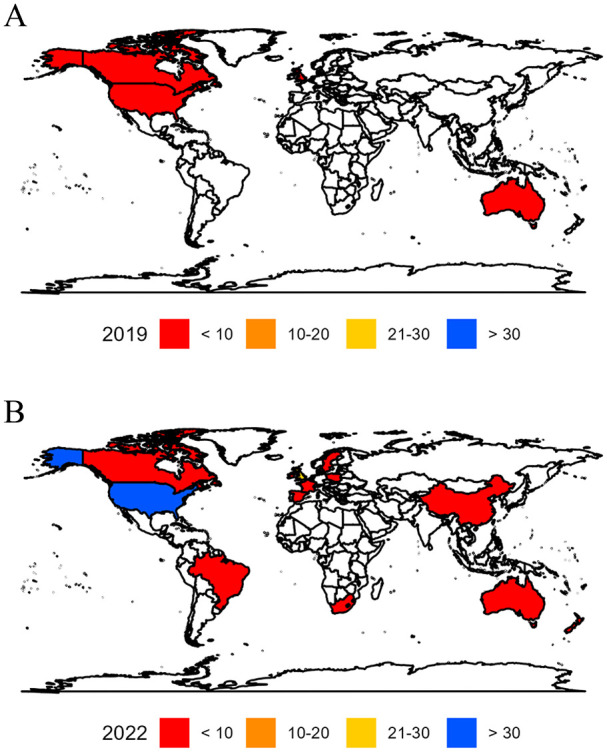
Geographical distribution of community involvement across the globe in 2019 (Panel A) and in 2022 (Panel B).

Before implementation, the majority of community involvement was conducted in the United Kingdom (*n* = 8, 67%) and United States (*n* = 2, 17%). After implementation, this trend continued (United Kingdom: *n* = 26, 29%; United States: *n* = 31, 34%), with emerging studies from Australia (*n* = 10, 11%). Community involvement in other parts of the world remain under-represented (e.g. in 2022, there was one study (1%) from Asia and one (1%) from Africa; see Figure 4).

When the number of articles published from each country was accounted for, the United Kingdom showed a proportionally high number of studies reporting community involvement (2019: 42%; 2022: 60%), followed by Canada (2019: 17%; 2022: 67%), Australia (2019: 13%; 2022: 83%) and the United States (2019: 4%; 2022: 55%; see Table S9). In 2022, while Brazil, New Zealand and Spain have substantially fewer papers published in *Autism* (*n* = 1 each) than United States (*n* = 56), United Kingdom (*n* = 43) and Australia (*n* = 12), 100% of these studies reported community involvement. See Table S9 for a detailed breakdown of frequencies and percentages across different countries.

#### Research areas

Both before and after policy implementation, more than half of the research reporting community involvement focused on applied issues, such as services and supports (2019: *n* = 4, 33%; 2022: *n* = 32, 35%) and lifespan issues (2019: *n* = 2, 17%; 2022: *n* = 28, 31%; see Table 4). In stark contrast, very few studies in areas of basic research, such as genetic and environmental factors, involved the community (2019: *n* = 0; 2022: *n* = 1, 1%). To investigate whether these proportions are a reflection of the type of research published in *Autism*, we included the frequency (and percentage) of articles published in 2019 and 2022 broken down by research area (see Table 4). Research on genetic and environmental factors was rarely published in *Autism* in 2019 (*n* = 0) or 2022 (*n* = 2, 1%). Papers focused on biology represented just over one-fifth of papers published; only a few of these papers reported community involvement in 2019 (*n* = 2, 7%), but this proportion increased to almost one-quarter post-implementation (*n* = 9, 24%). The increase, however, was particularly pronounced for research on lifespan issues (2019: *n* = 2, 17%; 2022: *n* = 28, 76%) and services and supports (2019: *n* = 4, 16%; 2022: *n* = 32, 64%). See Table S10 for a detailed breakdown of frequencies and percentages based on level of participation.

**Table 4.** Areas of research involving community members before (2019) and after (2022) policy implementation.

Research area	*N (%)*			
	Community involvement present		All published articles	
	Before (*n* = 12)	After (*n* = 91)	Before (*n* = 116)	After (*n* = 167)
Screening and diagnosis	1 (8%)	5 (6%)	16 (14%)	10 (6%)
Biology^a^	2 (17%)	9 (10%)	28 (24%)	37 (22%)
Genetic and environmental factors	0 (0%)	1 (1%)	0 (0%)	2 (1%)
Interventions	1 (8%)	13 (14%)	29 (25%)	25 (15%)
Services and supports	4 (33%)	32 (35%)	25 (22%)	50 (30%)
Lifespan	2 (17%)	28 (31%)	14 (12%)	37 (22%)
Infrastructure and prevalence	2 (17%)	4 (4%)	4 (3%)	6 (4%)

a Includes topics on brain structure and function and cognitive studies (Office of Autism Research Coordination, 2019).

#### Type of research methods

Both before and after implementation, approximately half of the articles reporting community involvement used quantitative methods (2019: *n* = 6, 50%; 2022: *n* = 43, 47%) and about one-third used qualitative methods (2019: *n* = 4, 33%; 2022: *n* = 28, 31%). Interestingly, when all published articles regardless of the presence of community involvement were considered, we observed a greater proportion of qualitative studies involving the community both pre-implementation (*n* = 4 out of 12, 33%) and post-implementation (2022: *n* = 28 out of 37, 76%) relative to quantitative studies (2019: *n* = 6 out of 84, 7%; 2022: *n* = 43 out of 93, 46%; see Table 5).

**Table 5.** Areas of research involving community members before (2019) and after (2022) policy implementation.

Research methods	*N* (%)			
	Of articles that reported community involvement		Of all published articles	
	Before (*n* = 12)	After (*n* = 91)	Before (*n* = 116)	After (*n* = 167)
Qualitative	4 (33%)	28 (31%)	12 (10%)	37 (22%)
Quantitative	6 (50%)	43 (47%)	84 (72%)	93 (56%)
Mixed methods	2 (17%)	13 (14%)	7 (6%)	15 (9%)
Reviews	0	7 (8%)	13 (11%)	22 (13%)

#### Type of funders

The majority of articles reporting community involvement, both pre- and post-implementation, had received funding from governments (2019: *n* = 7, 58%; 2022: *n* = 53, 58%) and trusts and foundations (2019: *n* = 5, 42%; 2022: *n* = 26, 29%). Particularly in 2022, these articles represent more than half of all studies published in *Autism* supported by governments (2019: 7 out of 71, 10%; 2022: 53 out of 97, 55%) and trusts and foundations (2019: 5 out of 38, 13%; 2022: 26 out of 51, 51%). Notably, 22 out of 37 unfunded studies published in 2022 (59%) involved community members (see Table 6).

**Table 6.** Types of funders supporting studies that involved community members before (2019) and after (2022) policy implementation.

Types of funders	*N* (%)			
	Of articles that reported community involvement		Of all published articles	
	Before (*n* = 12)	After (*n* = 91)	Before (*n* = 116)	After (*n* = 167)
Government	7 (58%)	53 (58%)	71 (43%)	97 (41%)
University	1 (8%)	24 (26%)	28 (23%)	41 (17%)
Trusts and Foundations	5 (42%)	26 (29%)	38 (23%)	51 (22%)
Service providers	0	2 (2%)	4 (2%)	6 (3%)
Industry	1 (8%)	4 (4%)	4 (2%)	4 (2%)
None	1 (8%)	22 (24%)	22 (13%)	37 (16%)

## Discussion

This study sought to examine the impact of the implementation of a mandatory community involvement statement in the journal, *Autism.* We found a fivefold increase in the proportion of published papers reporting community involvement in their research between 2019 (pre-implementation) and 2022 (post-implementation) – to the extent that half of all papers published in *Autism* in 2022 reported some degree of involvement, mostly involving Autistic people and parents of Autistic children or adults. This increase is encouraging and suggests that autism researchers are beginning to take seriously the ever-growing calls from community members of ‘nothing about us, without us’. Two additional findings serve to corroborate this view. First, there was a shift in the degree of community involvement: while, in 2019, most research reported community involvement at the consultative level, in 2022, there was an increase in the diversity of community involvement, including an increase in those reporting approaches at the higher rungs of the participatory ladder, namely, co-production, community-led and community-controlled research. Second, post-implementation of the mandatory statement also saw an increase in the diversity of activities in which community members were involved, ranging from project inception to the dissemination of findings (see den Houting et al., 2021 for similar findings). These changes reflect a potentially important shift in the research process – with researchers sharing roles in key decision-making across the research lifecycle.

The striking increase in reporting of community involvement is consistent with Price et al. (2018), who reported a 10-fold increase in the frequency of reporting of PPI following implementation of a mandatory statement in *The BMJ*, the first journal to introduce such statements. Notably, the absolute number of articles reporting such involvement is substantially higher in our study (*n* = 91, 54%) than in Price et al.’s (*n* = 16, 11%). This discrepancy is unlikely to be due to methodological differences: both studies adopted an inclusive approach, generally coding for the presence of community involvement, unless authors explicitly stated otherwise. Instead, it is possibly a result of variations in when the studies were conducted: Price et al.’s post-implementation sample was 2015–2016, 6 years earlier than our sample (2022). There is growing enthusiasm for, and expansion of, participatory approaches in research more broadly (Palmer et al., 2019; Tan et al., 2024), demonstrated in part by the emergence of a plethora of ‘co’ words (e.g. co-design, co-creation, etc.) – a phenomenon termed ‘cobiquity’ by some authors (Masterson et al., 2022; Williams et al., 2020). The relatively high number of published articles reporting community involvement might be one consequence of this broader trend. It might also be due to the nature of the research submitted to, and eventually published, in *Autism* specifically, which is often applied in nature (Table 4). There have been concerted calls to include community members in more basic science (Nature, 2018; Pellicano, 2020), but community members will be more likely to be involved in research that they themselves prioritise or which has the opportunity to make an immediate and tangible difference to people’s lives (Pellicano et al., 2014b; Roche et al., 2021).

Participatory approaches seek to ensure that the experiential expertise, knowledge and resources from involved community members enhance the rigour and effectiveness of the research – but their effects can be more far-reaching, even going as far as mitigating deep-rooted power imbalances between ‘researchers’ and ‘the researched’ (Beresford, 2003; Rose, 2013; Turnhout et al., 2020). While the increases in community involvement, as reported herein, are promising, several aspects of our findings also give reasons to be cautious.

*First*, much of the reported community involvement – at pre- *and* post-implementation of the mandatory statement – occurred at the design-focused part of the research. This included activities such as providing feedback on questionnaires, interview schedules and other participant-facing information (study adverts, information letters and consent forms). Prior to the participatory zeitgeist (Masterson et al., 2022), this type of involvement might simply have been conceptualised as a mainstream component of rigorous research – a validity or ‘sense’ check during the piloting process – rather than as it is construed now, as community involvement. Community involvement during this stage in particular can also mean that the major decisions around research remain those made by professional researchers, not community members – therefore making such processes susceptible to the dangers of tokenism (Arnstein, 1969; Ocloo & Matthews, 2016). While we cannot be sure, of course, that the community involvement in the studies coded herein was restrictive, the existing literature does not give reason for optimism in this regard (den Houting et al., 2021; Pickard et al., 2022). Clearly, explicating precisely how community members influenced the design process of the research, including what changes were made following their feedback and why, is one potential way of ensuring the ‘difference between . . . the empty ritual of participation and having the real power needed to affect the outcome’ (Arnstein, 1969, p. 216).

Yet, *second*, we also found that such transparent reporting did not occur nearly enough. There was a substantial minority of published papers reporting community involvement that contained insufficient or overly vague information, to the extent that it prevented us from coding such involvement – and even in those that did provide some information, coding was often not straightforward. Our team repeatedly encountered statements that did not report who the community members were,^3^ how they were engaged, or the impact of their involvement; that tended to conflate community involvement with standard research practices, such as member checking in qualitative research (Price et al., 2018; Weschke et al., 2023) and piloting processes (as we note above); and that often failed to distinguish community involvement in broader initiatives (e.g. intervention development, priority setting) from involvement in the research study on which the paper focused.

*Third*, there was also some confusion over who ‘counted’ as a community member, with some including clinical academics and researchers who were previously practitioners as community members. Indeed, there was a notable increase in the proportion of professionals, including clinicians and healthcare providers, educators and other professionals, designated as community members following implementation of the mandatory statement (Table 2). The inclusion of professionals as community representatives in participatory research is a deeply contested issue (Williams et al., 2020). While professionals contribute a particular sort of (clinical or practical) expertise and are often located outside of formal academic institutions, they are more distal in their relationship to the research than Autistic community members and their families. Also, just like researchers and policymakers, at least some practitioners have long held the power to shape research processes – far more than most lay community members – and their inclusion in autism research, especially over and above more direct beneficiaries such as Autistic people and their families, may therefore do little to challenge power inequalities (Boveda & Annamma, 2023).

These less-optimistic aspects of our findings might be due to a lack of awareness or recognition of one of the stated objectives of participatory approaches, namely, to reduce unequal power relations and potentially correct the epistemic injustice that prioritises the insights of scholars and professionals over the broader community (Arnstein, 1969; Israel et al., 2005; Turnhout et al., 2020). The existing research suggests that, even within grant programmes that champion participatory approaches, autism researchers tend not to investigate explicitly the critical role of power relations in their research design and conduct – and, as a result, community members report feeling left out of the decisions-making processes around research (den Houting et al., 2021, 2022). Researchers also might find it difficult to relinquish control over the fundamental elements of their research, and thus struggle to share power meaningfully with community members (Pickard et al., 2022). Some even contend that involving people with ‘a vested interest in the research’ like Autistic people themselves goes against orthodox scientific approaches to knowledge production in which many autism researchers are trained (Pellicano et al., 2014a). Support and training are therefore needed for researchers and community members to understand participatory research. Further support and training are needed for researchers to feel more confident in appreciating community members’ distinctive expertise (Milton, 2014), in developing ‘epistemic fluency’ (Markauskaite & Goodyear, 2017), or the ability to flexibly combine different forms of expertise and different ways of knowing, and in developing skills to resolve differences.

One potential way of addressing the challenges we note above is to share best practices and lessons learnt – including for unsuccessful attempts at participatory research (Turnhout et al., 2020) – *and* to be more transparent in our reporting the details of community involvement. Autism researchers are increasingly embracing more transparent, open science approaches (Fletcher-Watson, Brook et al., 2021; Hobson et al., 2022, 2023; Munafò et al., 2017), which are one way of enhancing research quality and safeguarding ethical practices (Bottema-Beutel, 2023; Dawson & Fletcher-Watson, 2022), overcoming replication failures (Gernsbacher & Yergeau, 2019), and correcting mistrust in autism research (Botha, 2021; Maye et al., 2022; Tan, 2023). The benefits of open science extend to participatory approaches, where high-quality reporting should enable researchers to critically appraise the quality of such approaches. Assessing such quality was not one of our study aims – and, in the event that it was, it would have been impossible to apply critical appraisal criteria given the often-limited information provided (Weschke et al., 2023). More complete and consistent reporting of community involvement could be supported through the use of the GRIPP-2 (Guidance for Reporting Involvement of Patients and Public; Staniszewska et al., 2017) as a reporting tool (see Table S7), improved guidance for authors, peer reviewers and editors, and the relaxing of manuscript word count restrictions for sections/statements on community involvement (Weschke et al., 2023). In the context of autism research, such guidance should also address the need to protect the identity of Autistic people involved in research who are not publicly ‘out’ as Autistic or whose family members are not either due to personal (Oswald, 2024) or sociocultural and political (Cheng et al., 2023) circumstances.

Funders also have a key role to play in enabling high-quality community involvement in (autism) research, perhaps in part by monitoring how funded community involvement translates into practice (Price et al., 2018). Major research funders, including government funding agencies and councils, increasingly require applicants to demonstrate evidence of meaningful community involvement in the development of grant applications and in the ensuing research. It is noteworthy, though, that the proportion of government-funded research reporting community involvement in *Autism* did not change following implementation of the mandatory statement. Instead, there was an increase in the proportion of unfunded projects reporting such involvement. We suspect that at least some of this unfunded research is conducted by doctoral students and early-career researchers, which would be encouraging given the strong appetite for conducting participatory autism research in this group in particular (Pickard et al., 2022), and by Autistic researchers themselves. It is also possible that some of this unfunded research involved salaried professionals as community partners who were compensated through their professional roles. Nevertheless, caution is warranted since compensating community members for their contributions to the research, including their time, skills, experiences and knowledge, is an essential part of high-quality community involvement and serves to balance power dynamics between researchers and the community (Black et al., 2013).

Overall, the mandatory reporting of community involvement required by *Autism* represents a significant and progressive systemic change that advances participatory processes in autism research. However, the prevalence of unclear reporting indicates that the current guidelines provided by the journal (see Supplementary Material) need improvement. Specifically, the guidelines should encourage authors to report detailed information on the research processes involving community members, the ways in which community members influenced the research, and reflect on the strengths and limitations of their participatory approaches (see Staniszewska et al., 2017, for reporting PPI activities using GRIPP-2). Furthermore – and in the spirit of genuine community involvement – it will be especially important for the journal to co-produce such reporting guidelines with the community, as did *The BMJ* (The BMJ, 2018).

### Limitations

There are several limitations of our research. First, our findings are restricted to research published in one autism research journal, *Autism*, which means we cannot be sure that the increased reporting of community involvement in research articles is due to the introduction of the mandatory statement per se, or whether this pattern reflects a general increase in community involvement in autism research (Tan et al., 2024). While we could have examined reports of such involvement in published articles from another autism research journal, we decided against this approach since the various autism journals attract a somewhat different collection of articles (e.g. *Autism in Adulthood* focuses specifically on adulthood, *Molecular Autism* has a biological focus), therefore making it difficult to compare like-for-like. It is also possible that research involving community members is more likely to have been submitted to *Autism* due to its public commitment to community involvement (Fletcher-Watson, Bölte et al., 2021). Second, although we were clear only to count community involvement that had occurred in the research reported directly in the published articles (rather than involvement reported to have occurred in previous research), it is possible that our inclusive approach to coding (Price et al., 2018) led to an *overestimation* of the frequency of involvement.

## Conclusion

Notwithstanding these limitations, our research revealed an encouraging trend of greater community involvement in autism research following the implementation of a mandatory reporting statement by the journal, *Autism.* The extent and nature of the community involvement post-implementation suggest that we are witnessing more research that is carried out ‘with’ or ‘by’ community members rather than ‘to’, ‘about’ or ‘for’ them. High-quality, transparent reporting of community involvement should enable the impact of community involvement to be evaluated. More needs to be done, though, to ensure that remaining inequalities and epistemic injustices are effectively tackled, and that those who have been too frequently overlooked or ignored in the past are properly recognised in the future.

## Supplemental Material

sj-docx-1-aut-10.1177_13623613241275263 – Supplemental material for Reporting community involvement in autism research: Findings from the journal Autism Supplemental material, sj-docx-1-aut-10.1177_13623613241275263 for Reporting community involvement in autism research: Findings from the journal Autism by Diana Weiting Tan, Laura Crane, Tori Haar, Melanie Heyworth, Rebecca Poulsen and Elizabeth Pellicano in Autism

## Notes

1. While it is possible to involve community members in authoring editorials and commentaries, given the restricted word allowance for such formats (1000 words), such reporting was deemed as less feasible than research and review articles (6000 words).

2. We acknowledge the limitation of coding country based on the corresponding author’s location, particularly for multi-site and international studies. Nevertheless, we adopted this approach under the assumption that a corresponding author, often also the lead or senior author, is likely to hold decision-making power around whether a study has community involvement.

3. It is important to note that, while *Autism* encourages authors to state whether they are working from a position of lived experience, this is optional – for good reasons (Oswald, 2024).
