# Supplementary material for: Reporting community involvement in autism research: Findings from the journal Autism
Source: Autism. 2024 Sep 6;29(2):490–503. doi: 10.1177/13623613241275263 (PMC11816470; doi:10.1177/13623613241275263)
Supplement: sj-docx-1-aut-10.1177_13623613241275263 – Supplemental material for Reporting community involvement in autism research: Findings from the journal Autism [file sj-docx-1-aut-10.1177_13623613241275263.docx]

**Supplementary Materials**

***Autism*’s community involvement statement policy – relevant clause:**

**2.8.3 Community involvement**

Autism encourages research that is actively carried out ‘with’ or ‘by’ members of the Autistic and autism communities (rather than ‘to’, ‘about’, or ‘for’ them), often referred to as ‘co-production’, ‘participatory research’, ‘patient and public involvement’ or ‘integrated knowledge translation’.

We therefore now require authors to include a community involvement statement at the end of the Methods section for Research Reports, outlining whether autistic people or family members, community providers, policy makers, agency leaders or other community stakeholders were involved in developing the research question, study design, measures, implementation, or interpretation and dissemination of the findings. Community members should be duly acknowledged – as authors or in the acknowledgements section – depending on the extent and nature of their contribution. We recommend that authors follow the BMJ’s editorial guidelines for documenting how community stakeholders were involved in their research.

If community members were not involved in the study, authors should state this.

For more details about the reasoning behind this journal requirement, and editorial expectations of authors, please download this FAQs document (<https://journals.sagepub.com/pb-assets/cmscontent/AUT/Community-Involvement-Reporting-FAQ-1626698718.pdf>).

**Pre-registered codebook (for full protocol, see** <https://osf.io/h5g4c/>)

**Table S1.** Coding scheme for obtaining general article information.

| **Code** | **Description** | **Rationale** |
| --- | --- | --- |
| Type of article | - Research article - Short report - Review - Letter to the editors | To examine whether certain types of articles were more likely to engage in community involvement than other types. |
| Type of empirical work | - Qualitative - Quantitative - Mixed methods | To examine whether certain types of research designs were more likely to engage in community involvement than other types. |
| Type of review | - Narrative/Literature review - Scoping review - Systematic review - Meta-analysis | To examine whether certain types of reviews were more likely to engage in community involvement than other types. |
| Country | Country(-ies) where the study was conducted (not applicable for reviews) | To examine where studies that involved community members took place. |
| Research area/discipline | Classification came from the IACC Strategic Plan, adapted and employed in den Houting & Pellicano (2019).   - Screening and diagnosis - Biology - Genetic and environmental factors - Interventions - Services and supports - Lifespan - Infrastructure and prevalence | To examine whether certain research areas were more likely to engage in community involvement than other areas. |
| Funding information | Classification adapted from Price et al. (2018)   - Industry - Trust - Governmental - University - Service providers - None | To examine whether studies supported by certain types of funders were more likely to engage in community involvement than other types. |

**Table S2.** Coding scheme for obtaining information on community involvement.

| **Code** | **Description** | **Rationale** |
| --- | --- | --- |
| Community involvement statement | Did the authors include a Community Involvement Statement, as required by Autism journal since 2021.   - Yes, with subheading ‘Community Involvement’ - Yes, with other subheading - No | To examine the frequency in which community involvement statements were included in articles published in *Autism*. |
| Presence of community involvement | Was there community involvement in this article?   - Yes, there was community involvement - No, there was no community involvement | To examine the frequency in which the community was involved in the articles published in *Autism*. |
| Excerpts of community involvement | Excerpts of community involvement will be extracted. | To provide contexts under which community involvement was undertaken. |
| Community members | Who were involved in this research?   - Autistic community members – adults - Autistic community members – adolescents and children - Autistic researchers* - Researchers* who are also parents/caregivers of autistic people - Researchers* who are part of the Autism community other than being parents/caregivers of autistic people - Parents/Caregivers of autistic adults - Parents/Caregivers of autistic adolescents and children - Other family members of autistic adults (e.g., grandparents, siblings) - Other family members of autistic adolescents and children (e.g., grandparents, siblings) - Clinicians/Healthcare providers - Educators - Policymakers - Other   * Researchers refer to people/academics who usually work within academic establishments or research institutions. | To examine the frequency in which certain groups of community members were involved in the research. |
| Breadth of community involvement | What did community members do?  Classification was adapted from Khodyakov et al. (2013) and den Houting et al. (2021).   - Developing community-based theories of the research - Grant proposal writing - Choosing research method - Designing study* - Developing sampling procedures - Recruiting study participants - Designing/Modifying interview schedules and/or survey questions - Implementing the intervention - Collecting primary data - Analysing data - Interpreting study findings - Writing reports/journal articles - Giving presentations at meetings/conferences - Insufficient information to determine breadth of involvement | To examine the frequency in which certain aspects of a research project involved community members. |
| Level of participation | Classification was based on Arnstein (1969)   - Community-controlled - Community-led - Co-producing - Engaging - Consulting - Informing - Educating - Coercing - Insufficient information to determine level of participation | To examine the frequency in which community involvement was conducted at each level of participation. |

**Documentation of data processing procedure**

**Table S3.** This table shows how we have handled information coded verbatim for breadth of involvement.

| **Direct quotes** | **Codes** |
| --- | --- |
| “Input into topics for research”  “Consulted on the aims of the study”  “Defining the objective of the metasynthesis”  “Study questions”  “Creating research questions and hypotheses”  “Developing hypotheses”  “Research questions” | **Pre-specified code:** Background research |
| “Co-developing research materials including the consent forms, fliers;”  “Providing feedback on the participant information sheets/participant-facing information”  “Giving feedback on study materials” | **Newly introduced code:** Providing inputs on public-facing materials |
| “Community members provided letters of support to grant agency” | **Newly introduced code:** Providing endorsement on project |
| “Reviewing manuscript” | **Pre-specified code:** Writing report/journal articles |
| “Actively involved in each phase of the study”  “Part of the research team throughout the study” | **Newly introduced code:** All aspects of research |
| “Piloted with parents of autistic and typically developing children to assess accessibility of the language and content of the form as well as informal evaluation of the question formats” | **Pre-specified code:** Designing/Modifying interview and/or survey questions |
| “Validating results”  “Study results were shared with panel members for feedback” | **Pre-specified code:** Interpreting study findings |
| “Dissemination of findings”  “Knowledge translation”  “Taking the lead in conveying the research findings to a wider autism community in China” | **Newly introduced code:** Disseminating findings (approach unspecified) |
| “Developing intervention”  “Developing training materials”  “Inputting into intervention”  “Redesigning intervention”  “Developing training”  “Creating intervention”  “Designing the intervention”  “Cultural adaptation [of intervention]” | **Newly introduced code:** Developing/Adapting intervention |

**Table S4.** This table shows how we have handled information coded verbatim for community members.

| **Direct quotes** | **Codes** |
| --- | --- |
| “Patient/Advocacy organisations”  “Advocacy organisation” | **Newly introduced code:** Patient/Advocacy organisations |
| “Practitioners”  “Professionals with a background in the research topic”  “Coaches” | **Newly introduced code:** Professionals (unspecified specialty) |
| “AAC specialist”  "Community providers and agency leaders“  “The implementation organisation” | **Pre-specified code:** Clinicians/Healthcare providers |
| “Community members”  “Community stakeholders”  “Supporters of autistic people” | **Newly introduced code:** Community members (unspecified connection) |
| “Caregivers”  “Relatives”  “Families with autism”  “Family members”  “Parents” | **Newly introduced code:** Family members (unspecified connection) |
| “Funding agency” | **Newly introduced code:** Funders |
| “Parents of autistic individuals”  “Parents of autistic people”  “Parent who had experience with the autism referral pathway” | **Newly introduced code:** Parents/Caregivers of autistic people (unspecified age) |

**Table S5.** This table shows how detailed coding scheme was transformed to a broad coding scheme for the breadth of involvement.

| **Detailed coding scheme** | **Broad coding scheme** |
| --- | --- |
| Developing community-based theories | Conceptualisation |
| Grant proposal writing |  |
| Background research |  |
| Providing endorsement on project |  |
| Choosing research method | Design |
| Designing study |  |
| Developing sampling procedures |  |
| Designing/Modifying interview schedules and/or survey questions |  |
| Providing inputs on public-facing materials |  |
| Developing/Adapting intervention |  |
| Recruiting study participants | Implementation |
| Implementing intervention |  |
| Collecting primary data |  |
| Analysing data | Analysis |
| Interpreting study findings |  |
| Writing reports/journal articles | Dissemination |
| Giving presentations at meetings/conferences |  |
| Disseminating findings (approach unspecified) |  |
| All aspects of research | Insufficient information |
| Insufficient information |  |

**Table S6.** This table shows how detailed coding scheme was transformed to a broad coding scheme for the groups of community members.

| **Detailed coding scheme** | **Broad coding scheme** |
| --- | --- |
| Autistic community members – adults (renamed as: Autistic adults) | Autistic people |
| Autistic community members – adolescents and children (renamed as: Autistic young people) |  |
| Autistic researchers |  |
| Parents/Caregivers of autistic adults (renamed as: Parents of autistic adults) | Families of autistic people |
| Parents/Caregivers of autistic adolescents and children (renamed as: Parents of autistic young people) |  |
| Other family members of autistic adults (renamed as: Family members [unspecified connection]) |  |
| Parents of autistic people (unspecified age) |  |
| Researchers who are also parents/caregivers of autistic people (renamed as Researchers who are also parents of autistic people) |  |
| Clinicians/Healthcare providers | Professionals |
| Educators |  |
| Professionals (unspecified specialty) |  |
| Researchers who are part of the Autism community other than being parents/caregivers of autistic people (renamed as: Researchers who are also professionals) |  |
| Policymakers | Administration |
| Funders |  |
| Patients/Advocacy organisation |  |
| Community members (unspecified connection) | Cannot be determined |
| Cannot be determined |  |

**Table S7.** Authors’ review of their participatory approach based on Staniszewska et al.’s (2017) Guidance for Reporting Involvement of Patients and the Public Version 2 (Short Form)

| **1. Aim**  Report the aim of the study | The aim of this study was to understand the impact of *Autism*’s introduction of mandatory reporting of community involvement in January 2021. Specifically, we examined the nature and extent of community involvement in all articles published in *Autism* before (in 2019) and after (in 2022) the implementation of this reporting policy.  The involvement of Autistic researchers (TH, MH, RP) was deemed necessary for two main reasons:   1. This is a project about community involvement hence should include community involvement. 2. Autistic people should have a say in deciding whether or not certain participatory approach is inclusive. |
| --- | --- |
| **2. Methods**  Provide a clear description of the methods used for community involvement in the study | The study was first conceptualised by non-autistic researchers (DT, LC, EP). After a brief discussion over email to map out a broad plan for carrying out this project, TH was first invited to join this collaborative work who contributed to the design and testing of the coding schemes. MH and RP were subsequently invited to join the team and contributed to the coding of the articles alongside TH, DT, LC and EP.  Over a six-month period, the team met once a month over Zoom for two hours to discuss coding and resolve coding discrepancies. After coding was finished, the team met twice to discuss the study findings and their implications.  DT and EP gathered these discussion points and wrote the first draft of the manuscript which was commented on by LC, TH, MH, and RP. The team also jointly submitted an abstract to present their work at an international conference. |
| **3. Results**  Outcomes – Report the results of community involvement in the study, including both positive and negative outcomes | TH, MH, and RP contributed to the study in several ways, including:   - Designing the coding schemes (e.g., differentiating Autistic researchers from Autistic community members) - Coding articles - Contributing to discussions to resolve coding discrepancies - Interpreting results (e.g., highlighting the impact of vague reporting of community involvement on community’s trust in research) - Initiating important discussions around the principles and aims of participatory research, as well as direct beneficiaries of autism research - Providing feedback on drafts of manuscript |
| **4. Discussion**  Outcomes – Comment on the extent to which PPI influenced the study overall. Describe both positive and negative effects. | Autistic and non-autistic researchers involved in this study felt that our collaboration was genuine and effective whereby autistic inputs were embedded throughout all aspects of this research process. There were two positive outcomes of our collaboration.  First, our team is diverse in our backgrounds, including our locations (Australia and United Kingdom), our career stages (both early-career and established researchers), our neurologies (both Autistic and non-autistic), and our connections to the community (parents of Autistic young people and Autistic self-advocates)—our team believes that this is a real strength of our study as these positionalities contributed to our conceptualisation and interpretation of an important question of what counts as community involvement. One study has provided empirical evidence for the benefits of collaborative coding from a diverse team of researchers (Zreik et al., 2022), which include enabling greater inclusion, teamwork and improved analysis. This was consistent with our own experiences from this project.  Second, the involvement of Autistic researchers in this project meant that the team is continuously reminded of the power differentials between the research and lay communities; power differences that non-autistic researchers may otherwise be less mindful of.  Nevertheless, one limitation in our approach was the exclusion of Autistic lay community members in the study team. While Autistic researchers are a part of the Autistic community, their academic expertise and positions may mean that they hold some academic power that Autistic lay community members do not have access to (see Raymaker’s (2019) reflection). |
| **5. Reflections**  Critical perspective – Comment critically on the study, reflecting on the things that went well and those that did not, so others can learn from this experience. | This project has given us an opportunity to work collaboratively to critically evaluate community involvement in autism research. While the team could not think of anything that did not go so well, there were three factors that contributed to our positive experience working together. First, as our team comprises researchers, we shared a common understanding of how research collaboration work (e.g., expectation to meet certain deadlines for coding, meeting after-hours due to being in different locations), which facilitated our collaboration. Second, some of us already had shared relationships from collaborating on prior projects while others were new to working together. Thus, our pre-existing relationships led to a degree of trust that mitigated possible challenges that may come with working with new collaborators. Third, our team shares similar principles and beliefs about participatory approaches and frequently adopt inclusive research practices in our work. Our shared values in this regard also enhanced our experiences in this collaboration. |

**
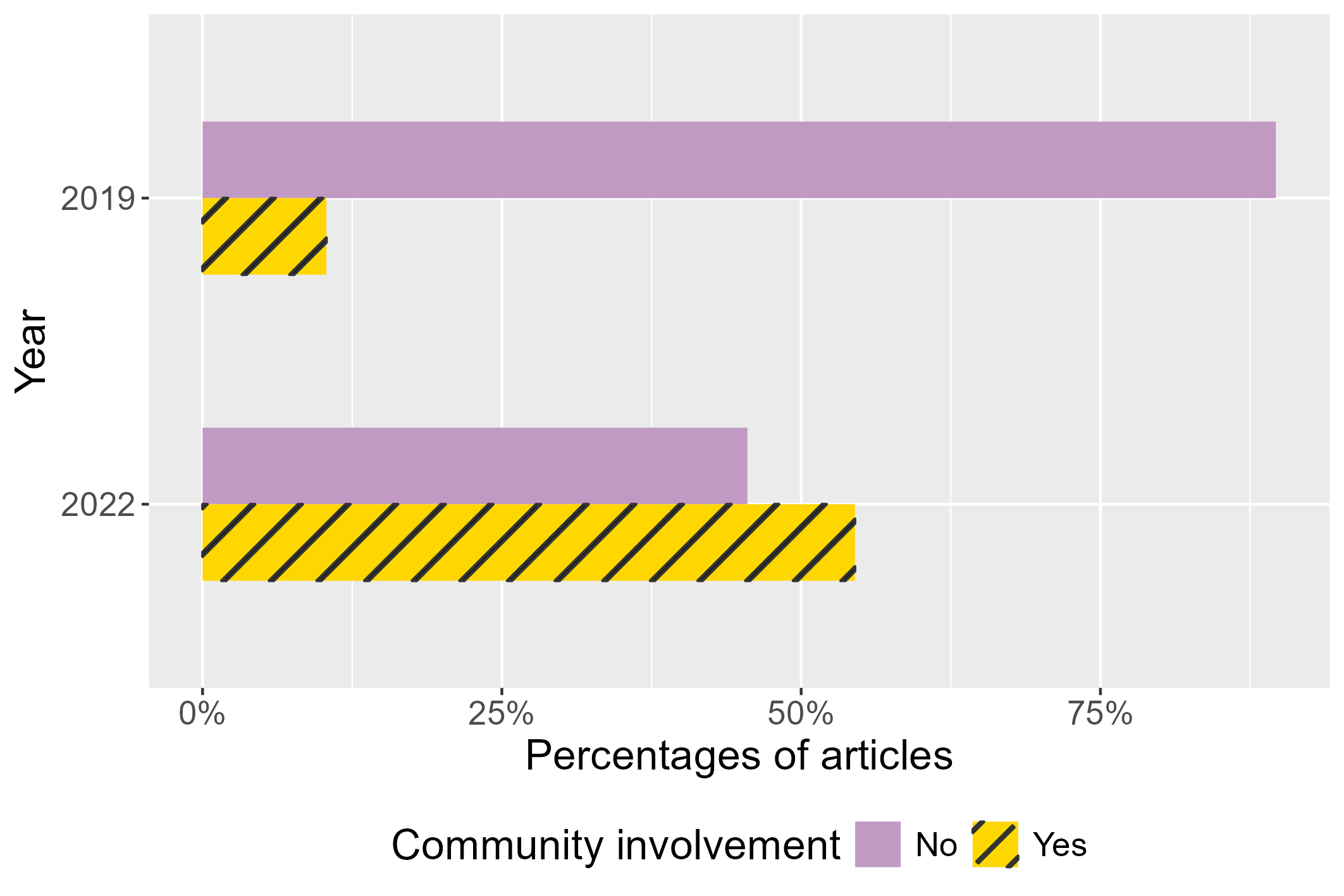
**

**Figure S1.** This bar chart shows the percentages of articles reporting the presence or absence of community involvement activities out of all eligible articles published in 2019 and 2022. Bar colours and patterns indicate presence of community involvement.

**Table S8.** Community involvement across different levels of participations and different groups of community members (based on the broad coding scheme) before and after policy implementation.

|  | ***N* (%)** | | | | | | | | | | |
| --- | --- | --- | --- | --- | --- | --- | --- | --- | --- | --- | --- |
|  | **2019 (*n*=12)** | | | | |  | **2022 (*n*=91)** | | | | |
| **Level of participation** | **Autistic people** | **Families** | **Professionals** | **Administration** | **Insufficient information** |  | **Autistic people** | **Families** | **Professionals** | **Administration** | **Insufficient information** |
| Comm controlled | 0 | 0 | 0 | 0 | 0 |  | 4 (4%) | 0 | 0 | 0 | 0 |
| Comm led | 0 | 0 | 0 | 0 | 0 |  | 5 (5%) | 1 (1%) | 2 (2%) | 0 | 0 |
| Co-producing | 2 (17%) | 0 | 0 | 0 | 0 |  | 15 (17%) | 11 (12%) | 6 (7%) | 2 (2%) | 1 (1%) |
| Engaging | 0 | 0 | 0 | 0 | 0 |  | 1 (%1) | 3 (3%) | 5 (6%) | 2 (2%) | 0 |
| Consulting | 4 (33%) | 3 (25%) | 2 (17%) | 1 (8%) | 0 |  | 19 (21%) | 13 (14%) | 10 (10%) | 2 (2%) | 2 (2%) |
| Informing | 0 | 0 | 0 | 0 | 0 |  | 1 (1%) | 0 | 0 | 0 | 0 |
| Insufficient info | 1 (8%) | 2 (17%) | 0 | 0 | 0 |  | 6 (7%) | 8 (8%) | 9 (9%) | 0 | 2 (2%) |

**Table S9.** Community involvement across different countries.

|  | ***N* (%)** | | | | |  | **% of articles with community involvement from each country** | |
| --- | --- | --- | --- | --- | --- | --- | --- | --- |
|  | **Of articles that reported community involvement** | |  | **Of all published articles** | |  |  |  |
| **Countries** | **2019**  **(*n*=12)** | **2022**  **(*n*=91)** |  | **2019**  **(*n*=116)** | **2022 (*n*=167)** |  | **2019** | **2022** |
| Australia | 1 (8%) | 10 (11%) |  | 8 (7%) | 12 (7%) |  | 13% | 83% |
| Belgium | 0 | 2 (2%) |  | 0 | 3 (2%) |  | 0 | 67% |
| Brazil | 0 | 1 (1%) |  | 0 | 1 (1%) |  | 0 | 100% |
| Canada | 1 (8%) | 6 (7%) |  | 6 (5%) | 9 (5%) |  | 17% | 67% |
| China | 0 | 1 (1%) |  | 1 (1%) | 5 (3%) |  | 0 | 20% |
| Denmark | 0 | 0 |  | 0 | 1 (1%) |  | 0 | 0 |
| Finland | 0 | 0 |  | 0 | 1 (1%) |  | 0 | 0 |
| France | 0 | 2 (2%) |  | 1 (1%) | 5 (3%) |  | 0 | 40% |
| Germany | 0 | 0 |  | 2 (2%) | 0 |  | 0 | 0 |
| Greece | 0 | 0 |  | 0 | 1 (1%) |  | 0 | 0 |
| India | 0 | 0 |  | 1 (1%) | 0 |  | 0 | 0 |
| Ireland | 0 | 1 (1%) |  | 0 | 0 |  | 0 | 50% |
| Italy | 0 | 0 |  | 4 (3%) | 2 (1%) |  | 0 | 0 |
| Israel | 0 | 0 |  | 2 (2%) | 2 (1%) |  | 0 | 0 |
| Japan | 0 | 0 |  | 2 (2%) | 1 (1%) |  | 0 | 0 |
| New Zealand | 0 | 1 (1%) |  | 1 (1%) | 1 (1%) |  | 0 | 100% |
| Poland | 0 | 1 (1%) |  | 1 (1%) | 2 (1%) |  | 0 | 50% |
| Portugal | 0 | 0 |  | 0 | 1 (1%) |  | 0 | 0 |
| Qatar | 0 | 0 |  | 1 (1%) | 0 |  | 0 | 0 |
| Singapore | 0 | 0 |  | 2 (2%) | 0 |  | 0 | 0 |
| South Africa | 0 | 2 (2%) |  | 2 (2%) | 3 (2%) |  | 0 | 67% |
| South Korea | 0 | 0 |  | 0 | 1 (1%) |  | 0 | 0 |
| Spain | 0 | 1 (1%) |  | 0 | 1 (1%) |  | 0 | 100% |
| Sweden | 0 | 2 (2%) |  | 2 (2%) | 4 (2%) |  | 0 | 50% |
| Taiwan | 0 | 0 |  | 0 | 3 (2%) |  | 0 | 0 |
| The Netherlands | 0 | 4 (4%) |  | 4 (3%) | 7 (4%) |  | 0 | 57% |
| UK | 8 (67%) | 26 (29%) |  | 19 (16%) | 43 (26%) |  | 42% | 60% |
| USA | 2 (17%) | 31 (34%) |  | 53 (46%) | 56 (34%) |  | 4% | 55% |

**Table S10.** Community involvement across different levels of participation and different research areas.

|  | ***N* (%)** | | | | | | | | | | | | | | |  |
| --- | --- | --- | --- | --- | --- | --- | --- | --- | --- | --- | --- | --- | --- | --- | --- | --- |
|  | **2019 (*n*=12)** | | | | | | |  | **2022 (*n*=91)** | | | | | | |  |
| **Level of participation** | **Screening & diagnosis** | **Biology** | **Genetic & environmental factors** | **Treatments & interventions** | **Services & supports** | **Lifespan issues** | **Infrastructure & prevalence** |  | **Screening & diagnosis** | **Biology** | **Genetic & environmental factors** | **Treatments & interventions** | **Services & supports** | **Lifespan issues** | **Infrastructure & prevalence** | |
| Community  controlled | 0 | 0 | 0 | 0 | 0 | 0 | 0 |  | 0 | 0 | 0 | 1 (1%) | 0 | 2 (2%) | 1 (1%) | |
| Community led | 0 | 0 | 0 | 0 | 0 | 0 | 0 |  | 0 | 0 | 0 | 0 | 2 (2%) | 4 (4%) | 0 | |
| Co-producing | 1 (8%) | 0 | 0 | 0 | 0 | 0 | 1 (8%) |  | 1 (1%) | 3 (3%) | 0 | 3 (3%) | 8 (8%) | 8 (8%) | 1 (1%) | |
| Engaging | 0 | 0 | 0 | 0 | 0 | 0 | 0 |  | 0 | 0 | 0 | 3 (3%) | 5 (6%) | 0 | 0 | |
| Consulting | 0 | 1 (8%) | 0 | 1 (8%) | 3 (25%) | 2 (17%) | 1 (8%) |  | 4 (4%) | 3 (3%) | 1 (1%) | 4 (4%) | 10 (10%) | 11 (12%) | 0 | |
| Informing | 0 | 0 | 0 | 0 | 0 | 0 | 0 |  | 0 | 0 | 0 | 0 | 0 | 1 (1%) | 0 | |
| Insufficient info | 0 | 1 (8%) | 0 | 0 | 1 (8%) | 0 | 0 |  | 0 | 3 (3%) | 0 | 2 (2%) | 6 (7%) | 2 (2%) | 2 (2%) | |
